# Alliin Attenuated RANKL-Induced Osteoclastogenesis by Scavenging Reactive Oxygen Species through Inhibiting Nox1

**DOI:** 10.3390/ijms17091516

**Published:** 2016-09-20

**Authors:** Yueqi Chen, Jingjing Sun, Ce Dou, Nan Li, Fei Kang, Yuan Wang, Zhen Cao, Xiaochao Yang, Shiwu Dong

**Affiliations:** 1 Department of Biomedical Materials Science, School of Biomedical Engineering, Third Military Medical University, Gaotanyan Street No. 30, Chongqing 400038, China; chenyueqi1012@sina.com (Y.C.); Sjjhx2012@163.com (J.S.); lance.douce@gmail.com (C.D.); li_nan1900@126.com (N.L.); kangfeilove2007@126.com (F.K.); wangyuancxh@163.com (Y.W.); zhenyacy@163.com (Z.C.); xcyang@tmmu.edu.cn (X.Y.); 2 Student Camp Four, Third Military Medical University, Chongqing 400038, China

**Keywords:** alliin, osteoclast, reactive oxygen species, Nox1

## Abstract

The healthy skeleton requires a perfect coordination of the formation and degradation of bone. Metabolic bone disease like osteoporosis is resulted from the imbalance of bone formation and/or bone resorption. Osteoporosis also reflects lower level of bone matrix, which is contributed by up-regulated osteoclast-mediated bone resorption. It is reported that monocytes/macrophage progenitor cells or either hematopoietic stem cells (HSCs) gave rise to multinucleated osteoclasts. Thus, inhibition of osteoclastic bone resorption generally seems to be a predominant therapy for treating osteoporosis. Recently, more and more natural compounds have been discovered, which have the ability of inhibiting osteoclast differentiation and fusion. Alliin (*S*-allyl-l-cysteine sulfoxides, SACSO) is the major component of aged garlic extract (AGE), bearing broad-spectrum natural antioxidant properties. However, its effects on bone health have not yet been explored. Hence, we designed the current study to explore its effects and role in receptor activator of nuclear factor-κB ligand (RANKL)-induced osteoclast fusion and differentiation. It was revealed that alliin had an inhibitory effect in osteoclasteogenesis with a dose-dependent manner via blocking the c-Fos-NFATc1 signaling pathway. In addition, alliin decreased the generation of reactive oxygen species (ROS) and down-regulated the expression of NADPH oxidase 1 (Nox1). The overall results revealed that alliin could be a potential therapeutic agent in the treatment of osteoporosis.

## 1. Introduction

Bone is an active, yet rigid, tissue with the capability of consuming a large amount of bone matrix, which also undergoes complex continuous remodeling, reshaping, and repairing [1]. In order to maintain bone homeostasis, osteoclastic bone-resorbing and osteoblastic bone-forming are needed to regulate the mineral balance and biochemical stability [2]. Osteoclasts are a specific polykaryon derived from monocyte/macrophage precursor cells/hematopoietic stem cells (HSCs) [3]. The most unique characteristic of osteoclasts is their ability of resorbing bone matrix by secreting hydrogen ions and lysosomal enzymes, such as cathepsin K and collagen dissolution protease. During the osteoclast differentiation and fusion, two of the critical factors are receptor activator of nuclear factor-κB ligand (RANKL) and macrophage colony stimulating factor (M-CSF) [4]. Osteoclasts play a predominant role in various skeletal diseases, such as postmenopausal osteoporosis, rheumatoid arthritis (RA), multiple myeloma (MM), cancer bone metastasis, and psoriatic arthritis (PsA) [5,6,7,8,9]. The binding of RANKL to its receptor induces small non-toxic amounts of reactive oxygen species (ROS), like various growth factors and cytokines, including tumor necrosis factor-α [10]. Moreover, reactive ROS can activate osteoclasts and enhance bone resorption [11].

Excessive generation of ROS usually results in oxidative stress, which plays a key role in the physiological processes of bone metabolism [12]. ROS and antioxidant enzymes are crucial signaling molecules in oxidative stress response and involved in metabolic bone diseases, such as osteoporosis caused by the imbalance of bone matrix [13,14]. During RANKL-mediated osteoclastogenesis, it was reported that Nox1 and mitochondria are key players, which disturb cellular redox homeostasis by creating oxidative stress through up-regulating ROS [13]. Taking ROS into consideration, the natural products with antioxidant activity could be novel medicines for treating bone metabolic diseases through decreasing ROS levels.

Several reports have demonstrated that many plant-based therapies for osteoporosis, including traditional Chinese medicine with potent antioxidant effects, might play an essential role in inhibiting osteoclast differentiation through their ability of scavenging ROS, such as genistein [15], baicalein [16], epigallocatechin-3-gallate [17], and fisetin [18]. Since alliin was discovered in 1947, it has been a well-known, biologically-reactive, non-protein, sulfur-containing amino acid of garlic [19]. Alliin was absorbed in the intestine through the transportation from amino acid to cysteine. Previous studies showed that alliin possessed a number of biological functions, including antibacterial, antimicrobial, anticholesterol, antiviral, antifungal, antioxidant activities, and other potential effects [20,21,22]. In some oxidant stress damage conditions, alliin could also act as an important inhibitor via controlling the generation of ROS and inhibiting the MAP kinase [22]. To our knowledge, the effect of alliin on bone health and the underlying mechanisms have not been investigated. Thus, it is meaningful for us to explore the effect of alliin on osteoclastogenesis.

In our study, we investigated that alliin inhibited RANKL-induced osteoclastogenesis due to its antioxidant characteristic. Furthermore, the ROS scavenging activity and generation regulated by alliin during the osteoclast differentiation and fusion in RANKL-induced osteoclastogenesis were examined.

## 2. Results

### 2.1. Alliin Has No Cytotoxicity on RAW264.7 Cells

**Figure 1:**
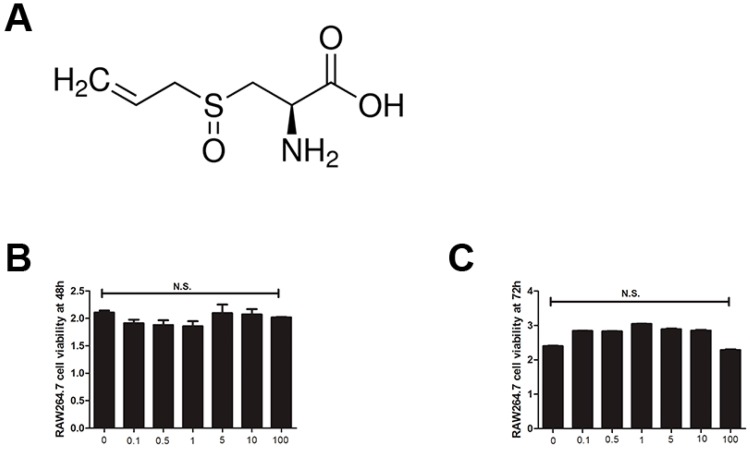
Alliin had no cytotoxicity during RANKL-induced osteoclastogenesis. (**A**) The chemical formula of alliin; (**B**) CCK-8 analysis of cell viability of RAW264.7 cells treated with various concentrations of alliin for 48 h; and (**C**) CCK-8 analysis of cell viability of RAW264.7 cells treated with various concentrations of alliin for 72 h. Data in the figures represent the mean ± SD. N.S. represents the difference was not statistically significant. It was based on one way ANOVA.

The chemical formula of alliin is shown in Figure 1A. In brief, the cytotoxicity of alliin was assessed by CCK-8 assay. RAW264.7 cells were treated with various doses of alliin with or without RANKL for 48 h or 72 h. As is shown in Figure 1B,C, alliin had no cytotoxic effects on cells at any doses after incubation. Interestingly, the viability of human umbilical vein endothelial cells (HUVECs) after exposure to SACSO at different concentrations (10–100 µM) for 24 h indicated that cells were not significantly affected [23]. Hence, in order to exclude the cytotoxic effects of alliin, non-lethal concentrations of alliin were chosen for the further subsequent experiments.

### 2.2. Inhibitory Effect of Alliin on Osteoclast Differentiation and Fusion in RANKL-Induced Osteoclastogenesis

**Figure 2:**
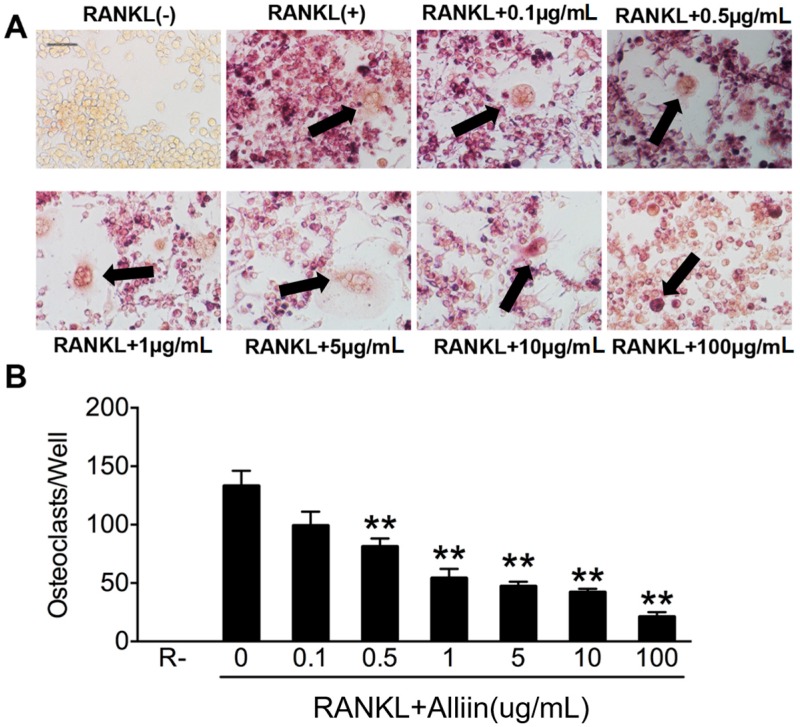
Alliin had an inhibitory effect on TRAP-positive cell formation. (**A**) Representative light microscope images of RAW264.7 cells cultured in a 96-well plate in the presence of RANKL (50 ng/mL) and M-CSF (50 ng/mL) with different concentration of alliin for 72 h were stained for TRAP (**red**). Scale bar represents 200 µm. The black arrows pointed out the representative TRAP (+) cells; and (**B**) quantitative analysis shows the number of TRAP (+) cells with more than three nuclei in each well (96-well plate). Data in the figures represent the mean ± SD. ** (*p*-value < 0.01) based on one way ANOVA.

In the next phase, Tartrate Resistant Acid Phosphatase (TRAP) staining was conducted to assess the effect of alliin on osteoclastogenesis. Osteoclast precursors (OCPs) were treated with different concentrations of alliin in the presence of RANKL (50 ng/mL) and M-CSF (50 ng/mL), and allowed to incubate into osteoclasts for three days. The morphological observations clearly demonstrated that the RAW264.7 cells differentiated into osteoclasts after adding alliin and RANKL was inhibited comparing to positive control. In order to determine whether alliin inhibits osteoclastogenesis in a dose-dependent manner, the count of multinucleated osteoclasts might reflect the TRAP activity. TRAP-positive cells were counted in each well of a 96-well plate, and photographed under a compound microscope. The increased TRAP action was associated with maturity in osteoclasts, was seen when OCPs were treated with RANKL. As is shown in Figure 2A,B, the number of TRAP-positive cells with three or more nuclei treated by different concentrations of alliin was decreased in a dose-dependent manner compared to the positive control. This exciting discovery suggested that alliin might inhibit osteoclasteogenesis in a dose-dependent manner.

**Figure 3:**
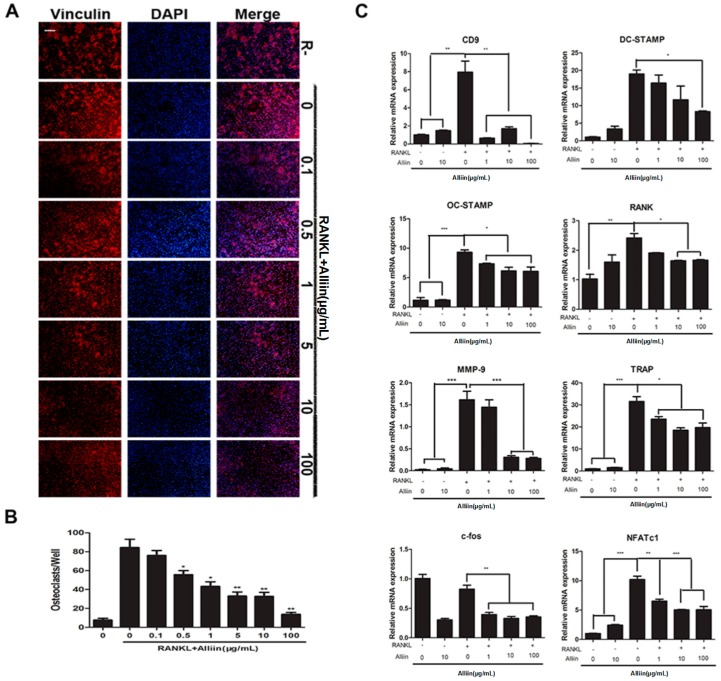
Alliin inhibited RANKL-induced osteoclast fusion and differentiation in a dose-dependent manner. (**A**) RAW264.7 cells were pretreated with RANKL (50 ng/mL) and M-CSF (50 ng/mL) for about 72 h along with a range of alliin concentrations (0, 1, 10 µg/mL), following focal and adhesion staining, and finally photographed. The nuclei were stained for double immunofluorescence microscopy by DAPI (**blue**) and Vinculin monoclonal antibody (**red**). Each experiment was performed thrice. Scale bar was at 200 µm; (**B**) the quantitative test for the TRAP (+) cells having multiple nuclei in each well of 96-well plate; and (**C**) the total RNA extracted from RAW264.7 cells during RANKL-induced osteoclastogenesis treated with RANKL (50 ng/mL) and M-CSF (50 ng/mL) for 72 h with varying doses of alliin (0, 1, 10, 100 µg/mL). Relative mRNA expression levels of NFATc1, c-Fos, MMP-9, CD9, DC-STAMP, OC-STAMP, TRAP, and RANK against GAPDH are shown. Data in the figures represent the averages ± SD. * (*p*-value < 0.05); ** (*p*-value < 0.01) or *** (*p*-value < 0.001) based on one way ANOVA.

In order to visualize the changes of osteoclast fusion, we performed actin cytoskeleton and focal adhesion staining and observed the mature osteoclast numbers and morphological characteristics of each osteoclast under a fluorescence microscope. Similar to the results of TRAP staining, the number of multinucleated osteoclasts was decreased (*p* < 0.01) (Figure 3A,B) and actin ring formation was inhibited after adding into various concentrations of alliin (≤100 µg/mL).

Based on these phenomena, the quantified confirmation of the inhibitory effect of alliin on RANKL-induced osteoclastogenesis was performed by quantitative real-time PCR. After we tested the expression of several important fusogenic genes and osteoclastogenesis specific genes at the transcription level, we found that the mRNA expression of 1 µg/mL, 10 µg/mL and 100 µg/mL alliin had a RANKL-induced inhibitory effect on osteoclast-related marker genes including TRAP, OSCAR, DC-STAMP, OC-STAMP, RANK, CD9, and MMP-9. The qRT-PCR data suggested that RANKLb up-regulated the expression on mRNA level of all the tested genes specifically compared to the non-treated controls, while alliin down-regulated expression of osteoclastic marker genes dose-dependently (Figure 3C). These results encouraged our idea that alliin diminished the RANKL-mediated the differentiation and fusion of osteoclasts.

### 2.3. Alliin Inhibits RANKL-Induced Osteoclastic Bone Resorption

**Figure 4:**
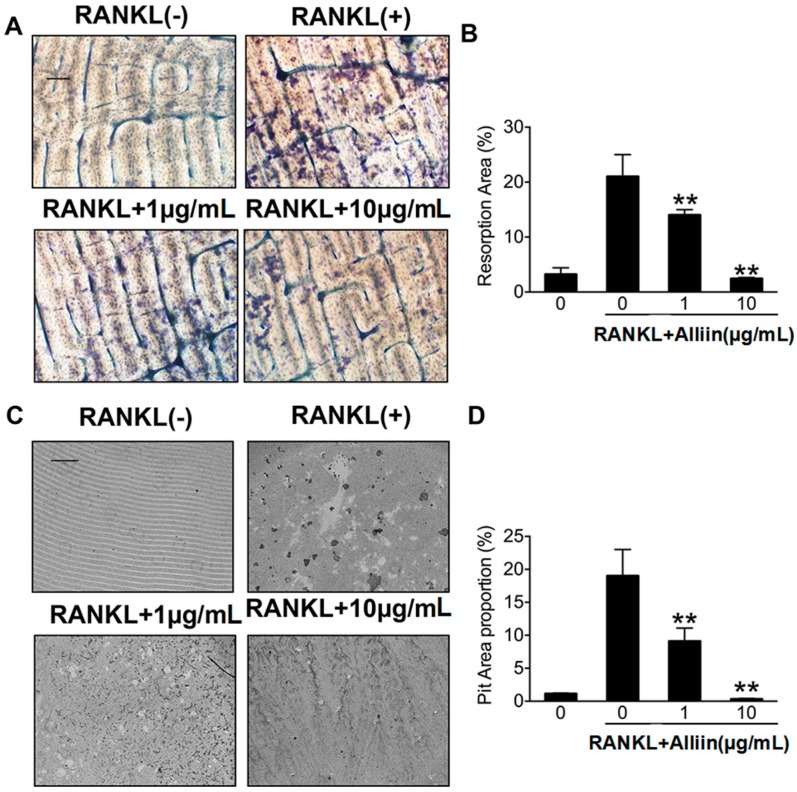
Alliin inhibited osteoclastic RANKL-induced bone resorption. (**A**) RAW264.7 cells cultured on bovine slices, were pretreated with RANKL and M-CSF (50 ng/mL each) for five days, along with a range of alliin concentrations (0, 1, 10 µg/mL), following focal and adhesion staining, and finally photographed. Experiments were done in triplicate. Scale bar represents 400 µm; (**B**) quantification of the bone resorption area on the bone slices; (**C**) representative images of RAW264.7 cells cultured on osteo assay surface 96-well plates treated with RANKL (50 ng/mL) and M-CSF (50 ng/mL) for five days with varying concentrations of alliin (0, 1, 10 µg/mL) followed removal of osteoclasts. Each experiment was performed thrice. The resorption area can be measured by 400 µm bars; and (**D**) measurement of the bone resorption area at the osteo surface. Data represented here at respective places is the averages ± SD. ** (*p*-value < 0.01) based on one way ANOVA.

Due to the exciting results of alliin on OCs fusion from TRAP stain assay and FAK stain assay, we then further hypothesized whether alliin has a similar function on bone resorption via pit formation assay. RAW264.7 cells were seeded onto bone slices and osteo assay surface and treated according to the assigned control. When they were incubated for five days, the medium was removed, the wells were dried and the resorption area was quantified. As is shown in Figure 4A,C, alliin (1–10 µg/mL) down-regulated osteoclastic bone resorption activity. Morphological observations were also made, and reduction in bone resorptive pits were confirmed by fluorescence microscopy. Quantitative analyses showed that the total bone resorption area was significantly decreased (*p* < 0.01) (Figure 4B,D). This discovery indicated that alliin could inhibit osteoclastic bone resorption activity because larger cell size and more multinucleated osteoclasts facilitated bone resorption.

### 2.4. The Inhibitory Effect of ROS Level and NADPH Oxidase Expression by Alliin during the Osteoclastic Differentiation of RAW264.7 Cells

**Figure 5:**
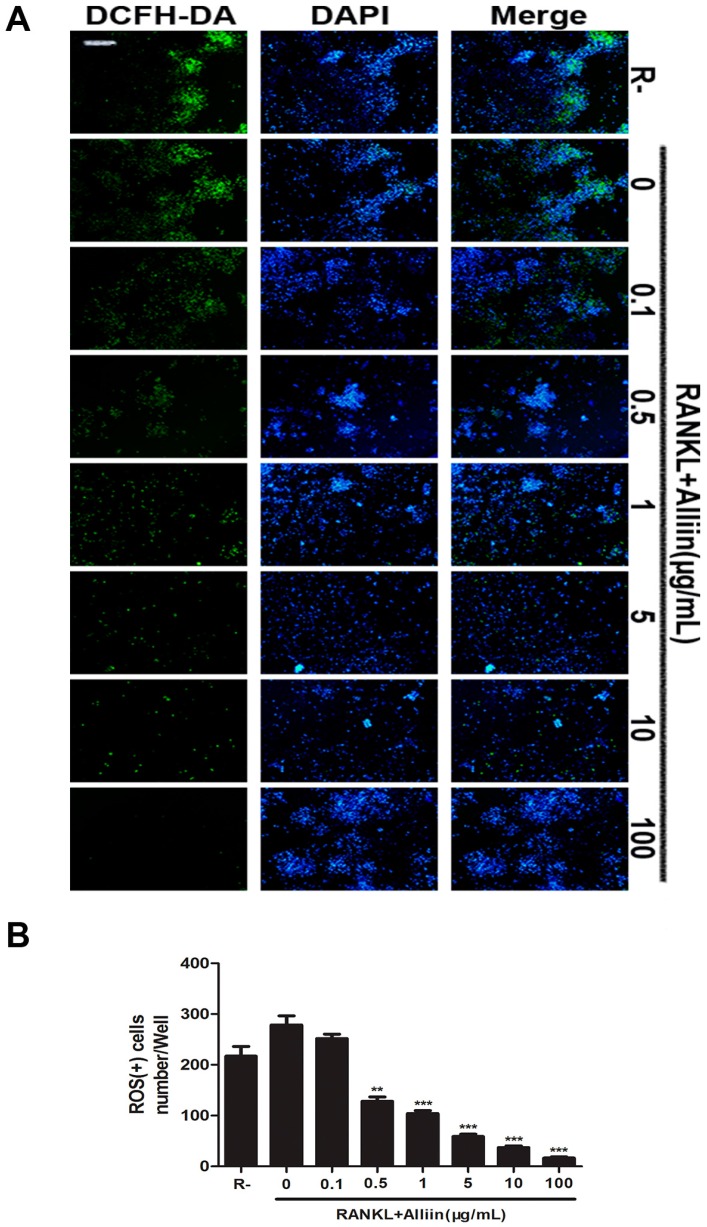
Alliin scavenged ROS production in a dose-dependent way. (**A**) ROS detection by fluorescent probe DCFH-DA. Scale bar represents 200 µm; and (**B**) quantitative analysis of ROS (+) cells in each well (96-well plate). Data in the figures represent the mean ± SD. ** (*p*-value < 0.01) and *** (*p*-value < 0.001) based on one way ANOVA.

**Figure 6:**
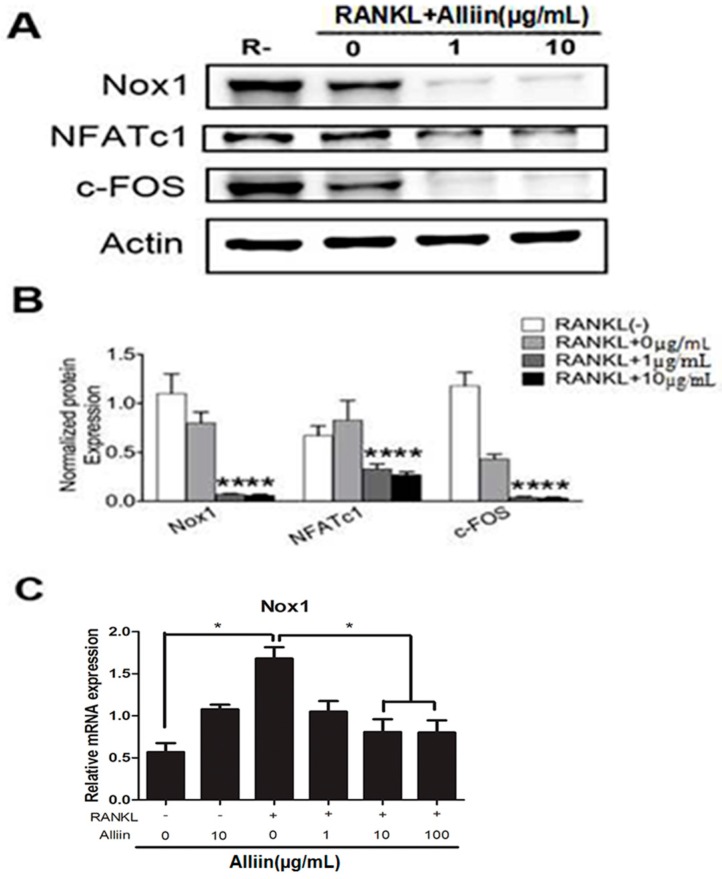
Alliin inhibited RANKL-induced osteoclast differentiation and fusion through inhibiting Nox1 activity and blocking the c-Fos-NFATc1 signaling pathway. Total protein was extracted from RAW264.7 cells during RANKL-induced osteoclastogenesis treated with RANKL (50 ng/mL) and M-CSF (50 ng/mL) for 72 h with varying doses of alliin (0 µg/mL, 1 µg/mL, 10 µg/mL). (**A**) Representative Western blot images of c-Fos, NFATc1, Nox1, and β-actin from RAW264.7 cells in different groups; (**B**) relative intensity of expression level of c-FOS, NFATc1, and Nox1 against β-actin; and (**C**) relative mRNA expression levels of Nox1 against GAPDH. Data in the figures represent the mean ± SD. * (*p*-value < 0.05); ** (*p*-value < 0.01) based on one way ANOVA.

It is known that ROS is correlated with RANKL-induced osteoclastogenesis and plays an essential role in bone resorption. In order to elucidate whether the beneficial effects of alliin on RANKL-induced osteoclastogenesis are linked with its ability of scavenging ROS, we measured ROS production using the cellular permanent oxidation-sensitive dye, DCFH-DA. Quantitative analysis showed alliin pretreatment significantly decreased the intracellular ROS level dose-dependently as compared to stimulation only with RANKL (*p* < 0.01) (Figure 5A,B). Moreover, it has been suggested that ROS-mediated RANKL-induced the fusion and differentiation of RAW264.7 cells into osteoclasts occurred through the activation of Nox homologues and the electron transport chain in mitochondria [20]. Additionally, reducing the activation of Nox1 has been reported to scavenge ROS upon RANKL-induced osteoclastogenesis [24,25,26]. Thus, in order to determine whether alliin suppresses superoxide anion production by Nox1, the transcription and activation level of Nox1 were analyzed by qPCR and Western blot. Consistently, the results showed that Nox1 expression was augmented by RANKL treatment alone. Alliin treatment markedly attenuated the RANKL-induced Nox1 expression in a dose-dependent manner, both in mRNA and protein levels (Figure 6A–C). These results clearly revealed that alliin could ameliorate the ROS-mediated osteoclastogenesis through inhibiting the expression of Nox1.

### 2.5. Alliin Inhibits RANKL-Induced Osteoclast Specific Transcription Factor Translation and Protein Expression

In osteoclast fusion and differentiation, positive modulation in RANKL, c-Fos and NFATc1 expression is of crucial importance, particularly the later is highly modulated by c-Fos. In addition, alliin effect osteoclastogenesis was confirmed by the expression of certain transcription factors such as NFATc1 and c-Fos. The qRT-PCR resuts confirmed that RANKL signficantly up-regulated the induction of NFATc1 and c-Fos, whereas alliin treatment significantly diminished the expression of these two specific transcription factors (*p* < 0.01) (Figure 3C). Furthermore, it was revealed that alliin significantly inhibited the expression of RANKL activated NFATc1 and c-fos expression at the protein level (*p* < 0.01) (Figure 6A,B). Both Western blot assay and qRT-PCR results depicted that alliin significantly diminished RANKL-mediated induction of the transcription factors c-Fos and NFATc1.

## 3. Discussion

Postmenopausal osteoporosis is one of the most important bone metabolic diseases, which is mainly attributed to excessive bone resorption caused by the insufficiency of estrogen. Estrogen plays an essential role in maintaining bone resorption and bone generation with its antioxidant effects in osteoclasts and stimulating effects on osteoblasts. To our knowledge, ROS stimulation is also an important factor that contributes to postmenopausal osteoporosis with estrogen deficiency [27,28]. ROS, mostly derived from mitochondrial activity, plays a crucial role in RANKL-induced osteoclast differentiation in murine bone marrow-derived macrophages. Our previous studies confirmed that alliin had strong antioxidant activity through inhibiting ROS generation [29]. Thus, these studies supported us to investigate the inhibitory effect of alliin in RANKL-induced osteoclast differentiation and formation via its capacity of scavenging ROS through inhibiting the expression of Nox1 and many potential mechanisms. Our research also showed that alliin could retard RANKL-induced osteoclast differentiation and formation without cytotoxicity.

According to the antioxidant activity assay of alliin on RANKL-stimulated RAW264.7 cells, the ROS production was remarkably decreased. In addition, alliin attenuated RANKL-induced osteoclast differentiation and formation from RAW264.7 cells by decreasing the number of multi-nucleic TRAP positive cells and inhibiting the expression of osteoclastic specific genes, such as TRAP, RANK, DC-STAMP, OC-STAMP, MMP-9, and other related transcription factors.

ROS usually acts as an essential mediator in apoptosis, proliferation, autophagy, and other cellular events. Consequently, the balance of intracellular ROS is predominantly determined by the generation and scavenging. NADPH oxidase of the Nox family are the predominant regulators of the generation of ROS [20,30]. Additionally, it has been reported that the activation of Noxhomologs play an important role in ROS production during the OCPs differentiated into mature osteoclasts by RANKL treatment [30,31]. Thus, alliin suppressed ROS generation, especially superoxide anions, via Nox1 during osteoclastogenesis first searched at transcriptional level. Interestingly, the activity of Nox1 is acutely inducible and requires an assembly with cytosolic subunits [31]. Coincidently, alliin inhibits the expression of Nox1 in a dose-dependent manner.

In summary, this study revealed that alliin could diminish RANKL-induced osteoclastogenesis and attenuate DC-STAMP, TRAP, and OC-STAMP expression via the c-Fos-NFATc1 signaling pathway. Additionally, alliin inhibited the production of ROS through the inhibition of Nox1. Alliin could potentially reduce ROS levels, which may result in the inhibitory effects on RANKL-mediated osteoclast fusion and differentiation. Furthermore, an applicable dose of alliin could possibly recover against multifarious bone disorders due to the imbalance of bone resorption and bone formation, such as osteoporosis. Thus, this novel effect of alliin could provide us a new insight into treating osteoclast-related bone disorders. It is also worth investigating the effect of alliin in vivo and, ultimately, verify through clinical trials.

## 4. Materials and Methods

### 4.1. Reagents and Antibodies

Alliin, which isolated from garlic (purity > 98%), was purchased from Sigma-Aldrich (St. Louis, MO, USA). Mouse macrophage cell line RAW264.7 cells were obtained from the American Type Culture Collection (Rockville, MD, USA). The RANKL and M-CSF were obtained from R & D Systems Inc. (Minneapolis, MN, USA). Cell Counting Kit-8 reagent was obtained from Dojindo Laboratories (Kumamoto, Japan). Dulbecco’s modified Eagle’s medium (DMEM) and Fetal bovine serum (FBS) were obtained from Gibco Life Technologies (Grand Island, NY, USA). Bovine cortical bone slices were obtained from Boneslices.com (Jelling, Denmark). Dichlorofluorescin diacetate (DCFH-DA) cellular ROS detection assay kits were obtained from Abcam (Cambridge, UK). Tartrate-resistant acid phosphatase (TRAP) stain kit was obtained from Sigma-Aldrich (St. Louis, MO, USA). Actin cyto-skeleton and focal adhesion staining kits were purchased from Millipore (Darmstadt, Germany). Penicillin-streptomy solution was obtained from Hyclone (Thermo Scientic, ‎Waltham, MA, USA). Antibodies specific for Nox1, NFATc1, c-Fos, and β-actin were purchased from Santa Cruz Biotechnology (Santa Cruz, CA, USA).

### 4.2. Totoxity Evaluation of Alliin on RANKL-Induced Osteoclastogenesis

In order to measure viability, equal numbers of RAW264.7 cells were seeded onto 96-well plates uniformly at 2 × 10^3^ cells per well, which were maintained in DMEM, supplemented with 10% FBS, and incubated in a humidified atmosphere of 5% CO_2_ at 37 °C. When cells had strongly adhered to the plates during the following days, the medium was removed. Fresh medium containing DMEM medium plus 10% FBS, 50 ng/mL RANKL, 50 ng/mL M-CSF, and different concentrations of alliin were added to each well, which were used to induce the osteoclastogenesis fusion and differentiation. Meanwhile, other plates were added only fresh medium containing DMEM medium and different concentrations of alliin. After cells were incubated for 48 or 72 h, the medium was removed and 100 μL Cell Counting Kit-8 (CCK-8) solution was added to each well. After 2-h incubation, the remaining fixed products were analyzed by using multi-detection microplate reader (BioTek Instruments, Winooski, VT, USA) at wavelength of 450 nm. According to the related protocol, the procedures were operated in turn. The wells only containing the CCK-8 reagent were used as blank controls.

### 4.3. Tartrate-Resistant Acid Phosphatase (TRAP) Staining Assay

In order to generate mature osteoclasts, RAW264.7 cells were placed onto 96-well plates (5 × 10^3^ cells/well) containing DMEM medium plus 10% FBS, 50 ng/mL RANKL, 50 ng/mL M-CSF, and different concentrations of alliin. After three days of induction, the medium was removed, and these cells were washed twice with phosphate buffered saline (PBS). Cells were fixed in 4% paraformaldehyde for 20 min and added TRAP staining solution containing 0.1 mg/mL of naphthol AS-MX phosphate, 0.3 mg/mL of Fast Red Violet LB stain, as directed by the manufacturer. Under a light microscope, TRAP-positive cells with more than three nulclei were counted as osteoclasts.

### 4.4. Actin Cytoskeleton and Focal Adhesion Staining

RAW264.7 cells were placed on 96-well plate (5 × 10^3^ cells/well) and incubated for 72 h. For actin cytoskeleton and focal adhesion staining, cells were washed twice with PBS, fixed for 20 min with 4% paraformaldehyde in PBS (PH 7.4) at normal temperature. Cells were then permeabilized in PBS containing 0.1% Triton X-100 for 5 min at room temperature, and then washed twice using PBS. Then, cells were washed by blocking buffer (1% BSA in PBS for 30 min). Cells were incubated for 1 h at room temperature with a primary antibody (Anti-Vinculin) diluted in blocking solution to a working concentration (1:300). This was followed by washing three times (5–10 min each) with washing buffer (0.05% Tween-20 in PBS). Cells were labeled for 1 h at room temperature with a secondary antibody (Alexa Fluor 488 Goat Anti-Mouse IgG (H + L) Antibody, Invitrogen, Carlsbad, CA, USA) (1:500), and TRITC conjugated Phalloidin (1:500) was diluted in PBS. Cells were then washed three times as above. Nuclei counter staining was performed by incubating cells with DAPI (1:1000) for 5 min at room temperature, followed by washing cells for three times with washing buffer and then mounted for fluorescence microscopy as previously described.

### 4.5. Resorption Pit Assay

One-hundred micrometer thick, 20 mm × 20 mm size slices of bone were gently cut from crushed bovine femoral compact bone. After washing with distilled water, the bone slices were stored in 75% alcohol. RAW264.7 cells were seeded onto a 48-well plate (1 × 10^4^ cells per well) and covered with bovine femoral bone slices. The culture medium of each well contained 10% FBS, 50 ng/mL RANKL, 50 ng/mL M-CSF, and different concentration of alliin. After five days of induction, the medium was removed and a 10% bleach solution was fixed in each well so as to detect the condition of pit formation. After incubating at 4 °C for 5 min, the wells were washed three times with distilled water. When the wells were dried, individual pits or multiple pit clusters were observed by using light microscope. The bone surface of the resorbed area was calculated by Image J software after staining with toluidine blue. Analysis was evaluated as described [32]. Three slices were used for analysis at each time point. These experiments were repeated at least three times.

### 4.6. Determination of Intracellular ROS

The level of intracellular ROS was determined by using the fluorescent probe, 2,7-dichlorofluorescein diacetate (DCFH-DA). In brief, the RANKL-induced RAW264.7 cells were grown and treated with different concentrations of alliin for 72 h. After the stimulation, the cells subsequently incubated with DCFH-DA (diluted to a final concentration of 10 μM) for 30 min at 37 °C in the dark. Meanwhile, nuclei staining was performed by DAPI (1:250) for 5 min at 37 °C (this is not the popular way to visualize nuclei in live cells). Then, these cells were washed twice with serum-free medium, and the relative intracellular ROS levels of fluorescence were quantified by a fluorescence microscope (Olympus, Tokyo, Japan).

### 4.7. RNA Extraction and Quantitative PCR Assay

Quantitative PCR was used to measure the expression of specific genes during osteoclastic formation. For real-time PCR, 1 × 10^5^ RAW264.7 cells were seeded in each well of a six-well plate and cultured in complete medium containing DMEM, 10% FBS, 100 U/mL penicillin, M-CSF (50 ng/mL), and RANKL (50 ng/mL). Cells were then treated with or without alliin (1, 5, 10, and 100 mg/mL) during the indicated time. Total RNA was extracted from RANKL-induced osteoclastogenesis by using Trizol reagent (Life Technologies). For qRT-PCR, single-stranded cDNA was biosynthesized from 1 ug total RNA using reverse transcriptase with oligo-dT primer according the manufacturer’s instructions (Promega, Madison, WI, USA). All reaction were carried out using SYBR Green Mix (Takara, Nojihigashi, Japan), and the PCR conditions for quantitative real-time PCR (qRT-PCR) were as follows: activation of enzyme at 94 °C for 5 min, 40 cycles of denaturation at 94 °C for 30 s, annealing at 55 °C for 30 s, and extension at 72 °C for 20 s. Quantitative RT-PCR was carried out using a CFX96 touch q-PCR system (Bio-Rad, Hercules, CA, USA). All reactions were run in triplicate and were normalized to the housekeeping gene *GAPDH*. The specific primers used for ampilication were as follows: *GAPDH*: forward, 5′-TCTGCTGGAAGGTGGTGGACAGT-3′ and reverse, 5′-CCTCTATGCCAACACAGTGC-3′; NFATc1: forward, 5′-CCGTTGCTTCCAGAAAATAACA-3′ and reverse, 5′-TGTGGGATGTGAACTCGGAA-3′; c-Fos: forward, 5′-AACAGATCCGAGCAGCTTCTA-3′ and reverse, 5′-GACTTTCCTGTCGAATGCACT-3′; OSCAR: forward, 5′-GGTCCTCATCTGCTTG-3′ and reverse 5′-TATCTGGTGGAGTCTGG-3′; RANK: forward, 5′-TCCAGCAGGGAAGCAA-3′ and reverse, 5′-GGGACACGGGCATAGA-3′; MMP-9: forward, 5′-ACCCGAAGCGGATT-3′ and reverse, 5′-GGCATCTCCCTGAACG-3′; DC-STAMP: forward, 5′-TTATGTGTTTCCACGAAGCCCTA-3′ and reverse 5′-ACAGAAGAGAGAGGGCAACG-3′; OC-STAMP: forward, 5′-GGGCTACTGGCATTGCTCTTAGT-3′ and reverse 5′-CCAGAACCTTATATGAGGCGTCA-3′; CD-9: forward, 5′-CGGTCAAAGGAGGTAG-3′ and reverse 5′-GGAGCCATAGTCCAATA-3′; TRAP: forward, 5′-CTTGTGGACGAAAATATGTGGCT-3′ and reverse 5′-GACTTTCCTGTCGAATGCACT-3′; Nox1: forward, 5′-ATGCCCCTGCTGCTCGAATA-3′ and reverse 5′-AAATTGCCCCTCCATTTCCT-3′.

### 4.8. Western Blotting

RAW 264.7 cells were seeded onto six-well plates (3 × 10^4^ cells/well) containing DMEM medium plus 10% FBS, and incubated for 72 h. For the fusion and differentiation of osteoclasts, the medium containing 50 ng/mL RANKL, 50 ng/mL M-CSF and different concentration of alliin. When the osteoclasts were observed under a microscope after the incubation, the medium was removed and cells were washed twice by PBS. Cells were lysed in radio immune precipitation assay (RIPA) buffer containing 10 mM Tris, pH 7.2, 150 nM NaCl, 5 mM EDTA, 0.1% SDS, 1% Triton X-100 and deoxycholic acid. The lysates were incubated on ice for 30 min and then centrifuged at 12,000 rpm for 10 min to precipitate the debris. The concentration of protein was measured by using a BCA protein assay kit. Thirty micrograms of each protein lysate were resolved by SDS-PAGE and the sample was transferred onto polyvinylidene difluoride (PVDF) membranes. After the membranes were blocked in 5% skim milk for 1 h, then they were incubated with rabbit antibodies at following dilutions (Nox1, 1:1000; NFATc1, 1:1000; c-Fos, 1:1000; β-actin, 1:1000) overnight at 4 °C and incubated with secondary antibodies (1:1000) for 1 h. β-actin was served as loading control. Antibody reactivity was detected by exposure in ChemiDoc XRS+ imaging system (Bio-Rad, Hercules, CA, USA).

### 4.9 Statistical Analysis

The quantitative data were measured at least three independent replicated of each experiment, which are also presented as the mean ± standard deviation (SD). Statistical differences were carried out by one-way ANOVA analysis of student’s *t*-test. All statistical analyses were performed using the statistical package SPSS (Statistical Package for Social Science, SPSS Inc., Chicago, IL, USA) program with “*” (*p* < 0.05), “**”(*p* < 0.01), or “***” (*p* < 0.001) defined as significant. N.S. represents the difference was not statistically significant.

## 5. Conclusions

This is the first study to report that alliin inhibited RANKL-induced osteoclastogenesis through scavenging ROS by inhibiting Nox1 and blocking the cFos-NFATc1 signaling pathway in a dose-dependent manner. The results suggested that alliin could be a potential therapeutic agent in the treatment of osteoporosis.
