# Correction: Chen et al. Alliin Attenuated RANKL-Induced Osteoclastogenesis by Scavenging Reactive Oxygen Species Through Inhibiting Nox1. *Int. J. Mol. Sci.* 2016, *17*, 1516

**DOI:** 10.3390/ijms27136055

**Published:** 2026-07-06

**Authors:** Yueqi Chen, Jingjing Sun, Ce Dou, Nan Li, Fei Kang, Yuan Wang, Zhen Cao, Xiaochao Yang, Shiwu Dong

**Affiliations:** 1 Department of Biomedical Materials Science, School of Biomedical Engineering, Third Military Medical University, Gaotanyan Street No. 30, Chongqing 400038, China; 2 Student Camp Four, Third Military Medical University, Chongqing 400038, China

In the original publication [1], there were mistakes in Figures 3A and 5A as published. Specifically, in Figures 3A and 5A, the authors inadvertently inserted fluorescent staining images during the final figure assembly. The authors have found each original raw data and made the corresponding corrections. The corrected Figure 3 and Figure 5 are shown below. The authors state that the scientific conclusions are unaffected. This correction was approved by the Academic Editor. The original publication has also been updated.

## Figures and Tables

**Figure 3:**
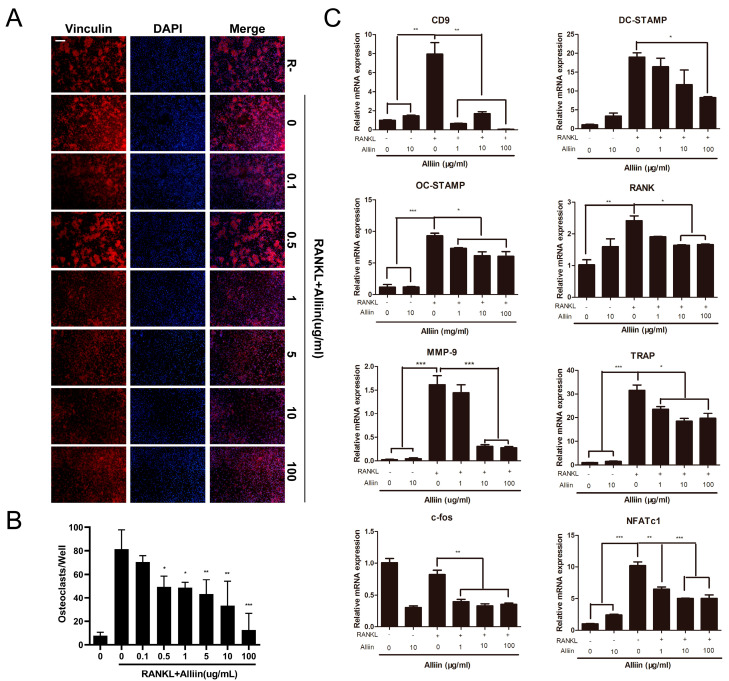
Alliin inhibited RANKL-induced osteoclast fusion and differentiation in a dose-dependent manner. (**A**) RAW264.7 cells were pretreated with RANKL (50 ng/mL) and M-CSF (50 ng/mL) for about 72 h along with a range of alliin concentrations (0 to 100 µg/mL), following focal and adhesion staining, and finally photographed. The nuclei were stained for double immunofluorescence microscopy by DAPI (blue) and Vinculin monoclonal antibody (red). Each experiment was performed thrice. Scale bar was at 200 µm; (**B**) the quantitative test for the TRAP (+) cells having multiple nuclei in each well of 96-well plate; and (**C**) the total RNA extracted from RAW264.7 cells during RANKL-induced osteoclastogenesis treated with RANKL (50 ng/mL) and M-CSF (50 ng/mL) for 72 h with varying doses of alliin (0, 1, 10, 100 µg/mL). Relative mRNA expression levels of NFATc1, c-Fos, MMP-9, CD9, DC-STAMP, OC-STAMP, TRAP, and RANK against GAPDH are shown. Data in the figures represent the averages ± SD. * (*p*-value < 0.05); ** (*p*-value < 0.01) or *** (*p*-value < 0.001) based on one-way ANOVA.

**Figure 5:**
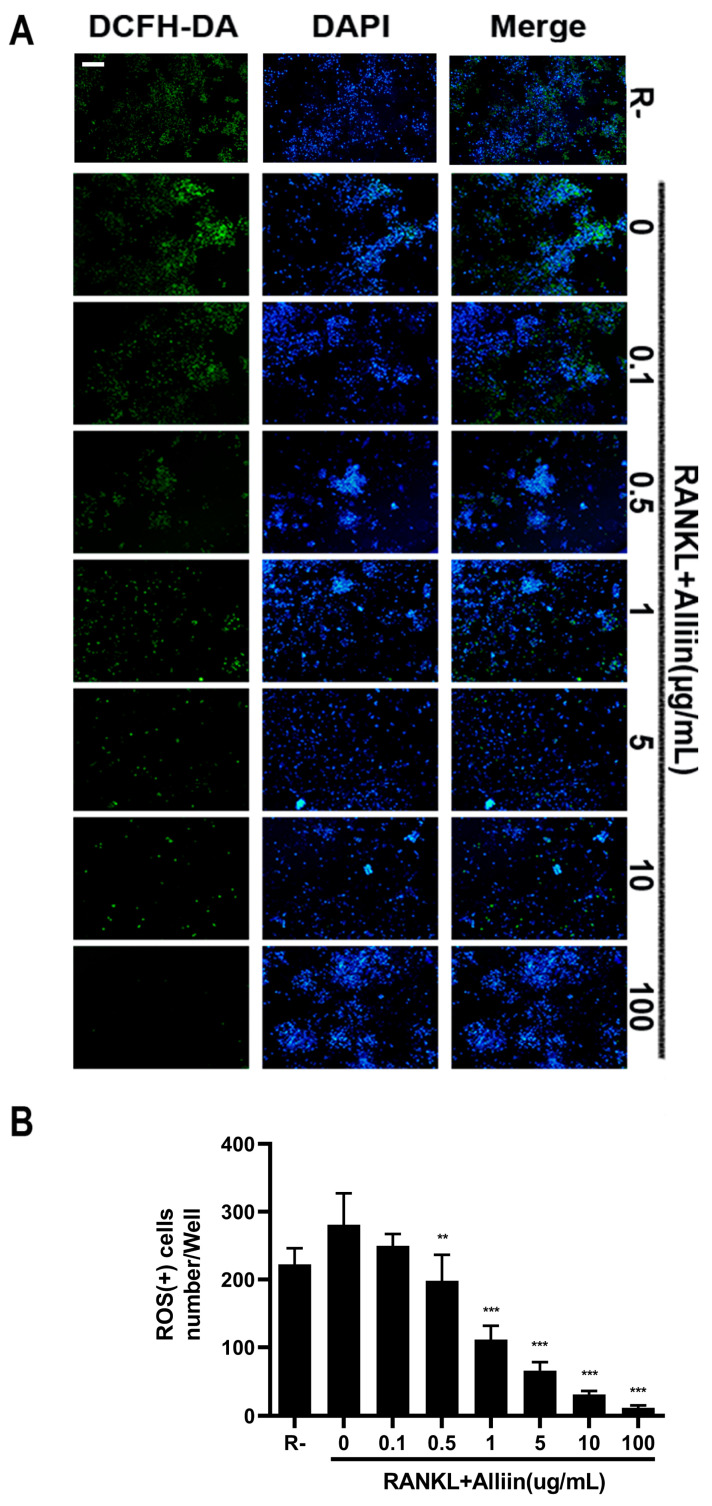
Alliin scavenged ROS production in a dose-dependent way. (**A**) ROS detection by fluorescent probe DCFH-DA. Scale bar represents 200 µm; and (**B**) quantitative analysis of ROS (+) cells in each well (96-well plate). Data in the figures represent the mean ± SD. ** (*p*-value < 0.01) and *** (*p*-value < 0.001) based on one-way ANOVA.
